# A prospective study on the characteristics and subjects of pediatric palliative care case management provided by a hospital based palliative care team

**DOI:** 10.1186/s12904-016-0166-8

**Published:** 2017-01-12

**Authors:** Charissa T. Jagt – van Kampen, Marijke C. Kars, Derk A. Colenbrander, Diederik K. Bosman, Martha A. Grootenhuis, Huib N. Caron, Antoinette Y. N. Schouten-van Meeteren

**Affiliations:** 1 Department of pediatric oncology, Emma Children’s Hospital, Academic Medical Centre, Amsterdam, Netherlands; 2 Department of medical humanities, Julius Center for health sciences and primary care, UMCU, Utrecht, Netherlands; 3 Department of pediatrics, Emma Children’s Hospital, Academic Medical Centre, Amsterdam, Netherlands; 4 Psychosocial Department, Emma Children’s Hospital, Academic Medical Centre, Amsterdam, Netherlands

**Keywords:** Pediatric palliative care, Case management, Anticipated care planning

## Abstract

**Background:**

Case management is a subject of interest within pediatric palliative care. Detailed descriptions of the content of this type of case management are lacking. We aim to describe the contents of care provided, utilization of different disciplines, and times of usage of a pediatric palliative care case management program compared for patients with malignant disease (MD) and non-malignant disease (NMD).

**Methods:**

A three-month prospective study, with questionnaires filled in by members of a pediatric palliative care team (PPCT) for each contact with parents.

**Results:**

Four hundred fifty-five contacts took place with parents of 70 patients (27MD, 43NMD). Sixty-two percent of all contacts were with the specialized nurse. The child life specialists, psychologist and social worker were also regularly consulted, the chaplain was not consulted. Ninety-five percent of all contacts took place between 8 am and 6 pm during weekdays, a limited number between 6 pm and 9 pm. Twenty-five percent of all contacts were proactively initiated by the PPCT, 25 % were initiated by parents. In these care characteristics, no differences were seen for MD and NMD patients. Psychosocial topics were addressed most frequently. MD patients consulted the PPCT more often about school and NMD patients about socio-economic issues.

**Conclusions:**

All different disciplines of the PPCT were regularly consulted, except for the chaplain. With an easy accessible team with a highly pro-active approach, availability from 8 am to 9 pm seems sufficient to accommodate patient’s and parent’s needs. More anticipation seems required for socio-economic topics. This insight in pediatric palliative case management can provide guidance in the development of a new PPCT.

## Background

Although pediatric palliative care (PPC) has not been standardized, the interest in PPC is increasing worldwide and a new WHO definition has been set [1]. New clinical practice guidelines have been developed and specialized pediatric palliative care teams (PPCTs) offering case management to children with life shortening disease are initiated [2–11]. Although an exact definition of case management is still under discussion, it should consist of anticipation of the care needed, and coordination of the multidisciplinary care process. Moreover it should be easily accessible, and include home visits, end-of-life planning, organization of respite care, and bereavement support [12]. Support by a PPCT can possibly result in fewer hospital admissions [10, 11], increased satisfaction with care [2, 5, 9, 13], better symptom management and quality of life [2, 8–10], and it will allow more patients to die at the preferred place [3, 6, 7]. However, studies investigating PPCTs often fail to specify the characteristics of care offered by each team [14]. Experts in a Dutch panel study specifically claimed that case management should be patient centered with a pro-active approach. However no consensus was reached on which disciplines should be part of the multidisciplinary approach, and on the need of 24-hour availability [15].

In the Netherlands, each year about 4200 children receive palliative care, which is delivered at home as much and as long as possible [16]. In June 2012 our University Children’s hospital initiated the first Dutch hospital-based multidisciplinary PPCT with specialized nurses experienced and trained in PPC, child life specialists, a psychologist, a chaplain and a social worker [17]. We recently presented a nine-month pilot study, concluding that patients with non-malignant disease (NMD) were supported for a longer period but less intensive (median 19 min/day, for 80 days) than patients with malignant disease (MD) (median 26 min/day, for 50 days) [17].

The aim of this current report is to gain detailed insight in pediatric palliative case management as provided by the PPCT, and the contents of contacts between the PPCT and parents. Since, in the pilot study, there were significant differences in duration and intensity of care between patients with MD and NMD, we chose to compare care characteristics between these patient groups. Because MD patients often have a shorter but more fulminant course compared to NMD, we assume to find differences also in the content of care needed by these families. We will specifically focus on the aspects of multidisciplinary approach, 24-hour availability, pro-active approach and finally on the topics of the contacts between the PPCT and parents.

## Methods

### Participants

Subject of study were all members of the PPCT, including the specialized nurses, the child life specialists, the psychologist, the social worker and the chaplain. The PPCT is located in a University children’s hospital in the capital of the Netherlands, and has the referral of patients below 19 years of age with any type of life shortening disease. The specialized nurses act as liaison case managers to organize PPC for the patient. The case managers coordinate the logistics of care delivered from all involved professionals. The PPCT has weekly multidisciplinary conferences to discuss patients. Two pediatricians and two pediatric oncologists are connected to the team, and join the weekly multidisciplinary conferences. Furthermore, the PPCT supports the first line professionals, patients and their families. The hospital based PPCT bridges the gap between hospital and home with contacts between parents and (primary) professionals. Contacts can be undertaken by telephone, mail as well as personal visits at home or during hospitalizations.

The primary physician remains responsible for the patient’s medical treatment and will introduce the PPCT to all patients with a life shortening disease, following any category as set by the WHO [1], early in the palliative phase of disease. The support is continuous during the course of illness and also provides bereavement support after the patient’s death. The PPCT’s support does not replace care, but is offered to navigate the patient through the complex care. The members of the PPCT registered data on their contacts with patients with any type of life shortening disease and their parents within their care between August 21^st^ 2013 and October 21^st^ 2013, and between April 8^th^2014 and May 8^th^ 2014. The second period was selected to be able to measure the need of support during a period with 5 extra holidays. Patient data including age, gender, disease characteristics, and number, duration and reasons for hospitalizations, were retrieved from the medical charts. Since the specialized nurses are available on every day of the week, for 24 h a day, we asked the members to specify, during what shift the contact took place.

### Study design

Any contact of a PPCT member with a patient or their parent - either by telephone, mail, visit at home or in the hospital - was subject for study. Since almost all contacts were with the parents of patients, in this report we will address all contacts as parent contacts. For data collection of this study all PPCT members prospectively completed a structured questionnaire on characteristics and content of each contact. Team members were prepared to work in a research setting and gave oral consent for participation. Furthermore, professionals were very specifically instructed on all issues of the form to tick boxes in the questionnaire addressing the following subjects: their discipline (specialized nurse, child life specialist, psychologist, chaplain, social worker), the initiation of the contact (planned = pre-arranged by the PPCT, parental = initiated by parents, and pro-active = unplanned pro-actively initiated contact by the PPCT), the time-shift of the contacts (office hours Monday through Friday between 8 am and 6 pm, evening/nights on weekdays, during weekends or national holidays), and the topics discussed during the contact from a list of 30 possible topics. One or more topics could be chosen per contact. For data-analysis these topics were categorized in 6 groups: physical, psychosocial, patient support (which includes patient support groups, patient counseling, and preparing/supporting medical procedures), school and daycare, socio-economic, and spare time, as is shown in Table 1. In the first version of the questionnaire, the identification of the discipline was printed too close to the paper margin, resulting in missing information. This aspect of the form was improved in the second study period and the importance of identification was explained to the team members.

**Table 1:** List of the six categories of 30 topics for contacts as reported by members of the PPCT

Physical	Pain/other physical symptoms Medication Toxicity Smoking/alcohol/drugs	Nutrition/fluids/sleep Appearance Self-support	Condition/fitness/energy Complementary support Sign language
Psychosocial	Emotions/feelings Relations with others Friendships/love affairs	Parents/siblings/family Stress reduction	Structuring the situation Balance in burden and resilience
Support patient/family	Patient organizations	Patient counseling	Preparing/supporting medical procedures
School/daycare	Remedial teaching/school support	Daycare	Cognition/learning/school
Socio-economic	Work Insurance	Leave Finances	Facilities
Spare time	Sports/relaxation	Respite care	Hobbies

Routinely a time registry of case management activities on each single patient was performed by PPCT members daily as part of their normal job, following a preset format to classify activities. Activities were divided in “time spent on direct contact with parents and/or patients”, and “other patient related activities”, which covered together the total time spent on individual case management. “Time spent on direct contact with parents and/or patients” included: intake interview, support at home and in the hospital, email and/or telephone contact, and aftercare. Time spent on “other patient related activities” included: consults with other disciplines, school or other involved professionals, multi-disciplinary conferences concerning a specific patient, and all supportive activities such as arranging equipment and medical aids, administrative activities and travelling time. Time spent on activities beyond individual care, but involving more than one patient, or team activities, such as the weekly multidisciplinary meetings, teaching and education, coordination and team support, were not included in this registration.

The Academic Medical Centre’s research ethics committee confirmed that the Dutch Medical Research Involving Human Subjects Act does not apply to the current study.

### Statistics

Since the patient cohorts, as well as the structure of the team were not substantially different during the two study periods, the results from both study periods were analyzed together as one study period of 89 days. We used descriptive statistics to present the majority of our results, such as number of contacts and time spent on contacts. Differences between patients with MD and NMD in intensity of support, number of contacts, initiation of contacts and time shift of contacts throughout the day, were assessed with a Kruskal Wallis test.

The distribution of topics was assessed for differences between patients with MD and NMD, different disciplines, time-shift of contacts, and different initiation of contacts. For each analysis of addressed topics an overall 2-tailed chi-square test was performed, and in case of significance, a chi-square was performed for each variable individually. For the variable difference in disciplines, those contacts without identification of the discipline were excluded from the analysis.

Data were analyzed in SPSS version 20, *p*-values <0.05 were considered significant.

## Results

During the study period of 89 days, the PPCT provided support to a total of 70 patients 27 with MD and 43 with NMD with characteristics shown in Table 2. At the beginning of the first study period 50 patients were already receiving support (19 MD, and 31 NMD patients). Eight patients were introduced to the team during the first study period (3 MD, and 5 NMD), one NMD patient died during this period. At the beginning of the second period, 40 patients (13 MD, 27 NMD) were receiving support, 3 patients (2MD, 1NMD) were introduced during the period. One MD patient died during the second period.

**Table 2:** Patient characteristics

Patients		MD 27		NMD 43	
Characteristics	Age (years) median (range)	10.2 (3.5–19.4)		4.0 (0.0–18.1)	
	Male/female	15/12		22/21	
	Disease	CNS tumor	11	Neuro- muscular	18
		Solid tumor	11	Congenital/syndromal	17
		Bone tumor	4	Metabolic	5
		Leukemia	1	Other	3
N and (median duration in days)		N (median)		N (median)	
Clinical admissions^a^		23 (3)		15 (8)	
PICU admissions		0		2 (1–10)	
Day hospital admissions^a^		57		5	
Patients admitted		14		13	
Indications for admission N	Anti-cancer therapy	59 (1)		0	
	Infection	6 (5.5)		11 (9)	
	Symptom treatment	5 (3)		6 (1)	
	Diagnostics	4 (1.5)		0	
	Transfusions	4 (1.5)		0	
	Other	2 (5.5)		2 (5.5)	
	Respiratory support	0		1 (8)	

Characteristics of the patient cohort of the PPCT, including hospital admissions and admission days, during 89 day study period

The disease group “other” in NMD consists of patients with chronic complex diseases: one with respiratory disease, one with gastro-intestinal disease and one with neurologic impairment as a complication of a herpes encephalitis

*Abbreviations: CNS* Central nervous system, *MD* malignant disease, *NMD* non-malignant disease

^a^Clinical admissions are hospital admissions with at least 1 night spent at the hospital. Day hospital admissions are hospital admissions with a duration of no more than one day

The time registry showed that the PPCT registered 526 activities with direct contact with parents, while 455 questionnaires were completed (87 % of contacts). 158 were from patients with MD and 297 from patients with NMD, with a median of 5 contacts per patient (range 0–37) (Table 3). Table 4 shows the structure of the PPCT, the number of professionals in each discipline, the number of contracted hours, as well as the number of hours registered for different aspects of case management.

**Table 3:** Time spent on support from the PPCT

Total study period	89 days
Duration support per patient; median (range)	60 days (9–89)
Number of contacts between PPCT and parents	526 of which 455 in questionnaires
Number of contacts per patient; median (range)	5 (0–37)
Patients with no registered contacts	6 (9 %)
Time spent by the PPCT members on case management	
- Total time spent on case management; median hours (range)	9.9 (0.3–87.5)
- Time spent on direct patient contacts^a^; median hours (range)	2.7 (0.0–20.3)
- Time spent per contact^a^; median minutes (range)	30 (5–195)
- Time spent^a^ per patient; median minutes (range)	225 (0–1400)
- Time spent on patient related activities: median hours^b^ (range)	6.2 (0.0–67.3)

Provides information on the duration and intensity of support from the PPCT. The information on contacts derives from the contacts as registered via the questionnaires of the team members. All contacts with any of the team members of the multidisciplinary PPCT are summed

The two categories together describe the total time spent on case management

^a^Time spent on “direct contacts” included intake interviews, support at home and in the hospital, email and/or telephone contact, and aftercare

^b^Time spent on “other patient related activities” include: consults with other disciplines, school or other involved professionals, multi-disciplinary conferences concerning a specific patient, and all supportive activities such as arranging materials, administrative activities and travelling time

**Table 4:** Parent and/or patient contacts and activities of the professionals of the PPCT

Function	Number of professionals	Contracted hours per week	Time registered for direct patient contacts (hours/week)	Time registered for patient related activities (hours/week)
Specialized nurse	5	122	20.8	39.8
Child life specialist	2	12	3.1	1.6
Psychologist	1	12	2.9	2.7
Chaplain	1	2	0	0
Social worker	1	4	1.5	0.4

Provides insight in the structure of professionals in our PPCT. The number of contracted hours per week is the sum of contracted hours of all professionals within each discipline. The time registered on different aspects of case management is the sum of registered hours spent on directly patient related case management for all professionals within each discipline, divided by the length of study period which is 12.7 weeks. Excluded in this registration is time spent on the general weekly multidisciplinary meeting, teaching and education, team support, research activities, coordination, absence of members due to vacation and/or illness, and other non-patient directed activities

**Fig. 1:**
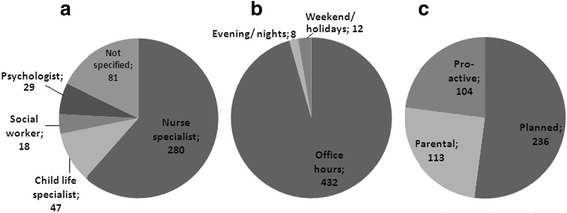
Provides an overview of the characteristics of all registered contacts the PPCT had with parents during the three month study period. **a** shows the distribution of contacts among the different disciplines. No contacts between parents and the chaplain were registered. **b** shows the distribution of contacts in different time shifts. The evening/night shift is defined as all hours between 6.00 pm and 8.00 am. Weekend and holidays during the study period included 24 weekend days and 4 Dutch holidays. **c** shows the distribution of contacts for different ways of initiation. The definitions of the different ways of initiation of a contact are: - planned = contacts that were pre-arranged between the PPCT and parents such as intake interview or follow-up on an earlier question, -parental initiative = a contact that was initiated spontaneously by the parent -pro-active = an unplanned contact initiated by the PPCT to inform how the patient is doing and to assess whether more support is needed

Contacts per discipline, time-shift of contacts, and initiation of contacts are shown in Fig. 1.

Of the 455 contacts between the PPCT and parents, 280 (62 %) contacts were with the specialized nurses, and a quarter of the contacts was with other members of the PPCT. No contacts were registered by the chaplain. For 80 (18 %) contacts the team member profession was missing due to inadequate lay out of the first version of the form (Fig. 1a). The chaplain confirmed that none of the unidentified forms derived from her.

Most contacts took place during office hours (95 %), and a limited number between 6 pm and 9 pm on working days. Ten patients (3MD, 7NMD) had 16 contacts during out of office hours (4 patients (1MD, 3 NMD) during evening/night, 8 patients (2 MD, 6NMD) during weekends/holidays). Fifteen telephone contacts were held, one home visit. Nine telephone contacts were initiated by the PPCT for follow up on earlier discussed topics, 6 telephone contacts were initiated by the parent or caregiver. Two of these contacts were about medication, two about the child getting ill, one about a possible retention bladder and one a planned follow-up on earlier discussed symptoms. The house visit was a multidisciplinary meeting with the oncologist, the primary physician, the home care and the PPCT to discuss the start of the palliative phase. This meeting was planned in the evening on purpose. None of the out of office hours contacts took place in the last week before death.

**Fig. 2:**
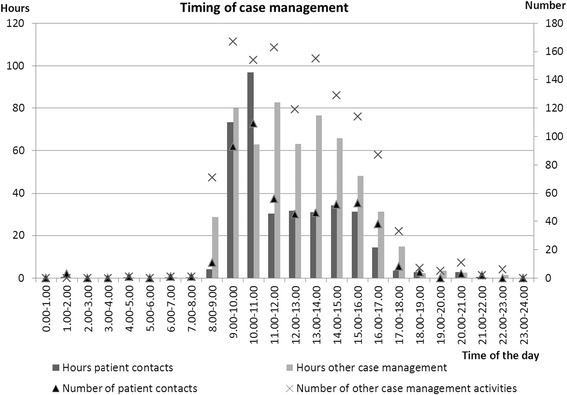
Timing of support provided by the PPCT throughout 24 h shift. Provides insight in the number, as well as the amount of hours the PPCT spent on parent contacts and other aspects of case management activities, at each time point during 24 h

A similar pattern was seen in the time registry, in time spent on case management, as is given for contacts and other activities of the PPCT spread over 24 h in more detail in Fig. 2.

Half of the contacts were planned by the PPCT, a quarter was initiated by parents, and the other quarter were unplanned contacts pro-actively initiated by the PPCT. The contacts initiated by the parents, and the pro-actively initiated contacts were mainly with the specialized nurse (88 and 92 % respectively). Sixty-one percent of the planned contacts were with the specialized nurse, 19 % with the child life specialist, 13 % with the psychologist, and 7 % with the social worker. No significant differences between patients with MD and NMD were found.

**Fig. 3:**
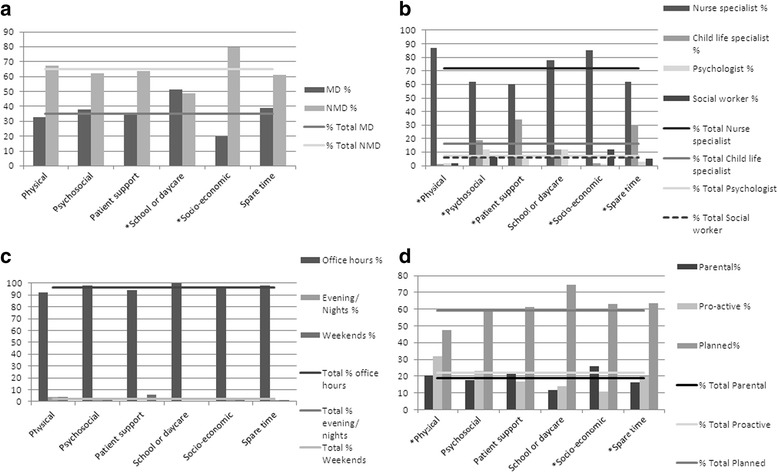
Topics of contacts with members of the PPCT. Provides insight in the distribution of discussed topics among different variables. The bars illustrate the percentage of total times a certain topic was discussed for each variable. The lines illustrate the percentage of all discussed topics for each variable. All topics with an asterisk show a distribution significantly in (dis) favor for at least one of the variables. **a** illustrates the distribution of topics among patients with MD and NMD. **b** illustrates the distribution of topics among different disciplines. **c** illustrates the distribution of topics among different moments on the day. **d** illustrates the distribution of topics among different initiations of contacts

A total of 672 topics were discussed with parents (234 MD, 438 NMD), of which 156 (23 %) were physical issues (51 MD, 105 NMD) and 257 (38 %) were psychosocial issues (97 MD, 160 NMD). There was an overall significant difference in distribution of topics for patients with MD vs NMD (Fig. 3a). Parents from children with MD, discussed school and daycare significantly more often (22/234 vs 21/438, *p* = < 0.001). Parents of NMD patients discussed significantly more socio-economic issues (80/438 vs 20/234, *p* = <0.001).

The distribution of topics among the members of the PPCT was also significant (Fig. 3b). In 544 (81 %) of the reported topics of discussion, the discipline was identified. Of these topics, 392 (72 %) were discussed by the specialized nurses, 88 (16 %) by the child life specialists, 34 (6 %) by the psychologist, and 30 (6 %) by the social worker and none by the chaplain. The specialized nurses handled 87 % of all physical topics, 62 % of all psychosocial topics, but also 85 % of all socio-economic topics. The specialized nurse handled physical topics and socio-economic topics more often than other topics. The child life specialist was consulted significantly more often for patient support and spare time issues, the psychologist for psychosocial issues and the social worker for socio-economic topics.

Physical topics were addressed significantly less often during planned contacts, while significantly more often in pro-active contacts initiated by the PPCT (Fig. 3d). Socio-economic issues were addressed significantly more often in contacts initiated by parents while less often in contacts pro-actively initiated by the PPCT. No significant differences were seen for the distribution of topics for different time-shifts (Fig. 3c).

## Discussion

This report provides detailed insight in the fulfillment of pediatric palliative case management by our PPCT with emphasis on direct parent contacts. We found that the specialized nurses are responsible for the highest proportion of contacts (62 %), and the psychosocial disciplines for about 25 %, although identification of the contacts was missing in 18 %. Most contacts (95 %) took place during office hours, and half of the contacts were initiated un-planned by either parents or the PPCT, especially those with the specialized nurses.

### Multidisciplinary approach

Although the multidisciplinary approach has been described in both adult and pediatric literature on palliative case management, there is no consensus on which disciplines should be present in the team. [2, 3, 5–7, 9, 10, 13] In our cohort the specialized nurse is the first person answering the phone and addressed for all different subjects, although significantly less often consulted on psychosocial issues, patient support issues and issues on spare time, indicating the need for psychosocial disciplines, which is in accordance with empowerment as felt appropriate for earlier training programs in the USA [18].

Spirituality is important in PPC, for patients as well as for parents [18–20] although, during our study period, the chaplain did not register any contacts. In multiple U.S. PPC programs, however, chaplains fulfill an active role in patient and family support, as well as in team support and education [21, 22].

Reports describing the involvement of chaplains in Dutch PPCTs are lacking [23].

Fewer chaplain contacts for Dutch patients could result from a different degree of families practicing a religion and/or that families preferably address their own minister or priest for religious support, which outweighs the contact with a chaplain from the hospital. Also, it might imply that other disciplines fulfill the need for spiritual support diminishing the need for the chaplain as an additional caregiver. The PPCT is aware of the availability of the chaplain since she is always present during the weekly meetings and works regularly on the different pediatric wards, despite being contracted for the PPCT for only 2 h a week. Future studies on the role of the chaplain regarding religion as well as spiritual care, team support and education are needed.

### 24- hour availability

Our PPCT offers 24-hour availability, like some other PPCTs [3, 6, 7, 9, 10]. However there is no consensus on the need for 24-hour availability [15]. Our results, show that the majority of contacts and time spent on case management is done between 8 am and 9 pm. Moreover, during the out of office hours only 6 telephone contacts were initiated by the parents for (semi) acute questions. The absence of requests for care during the late evenings and nights could be explained by the anticipating approach of the PPCT, ensuring that all needs are accounted for during daytime. Secondly the reliability of an adequate first line system in our country, including specialized pediatric home care, and easy access to tertiary care, are factors that might contribute to the low amount of consultations of the PPCT on duty. Strong first line support for PPC was also described in the UK West Midlands, although more collaboration with the medical specialist was preferred, while in the USA a quite similar supporting program Footprints bridges the gap between hospital and home [5, 24]. On the contrary in more rural infrastructures, for instance Australia, the amount of night shift calls for children with cancer reached up to 10 % [25]. In our Dutch health care system, a full 24-hour availability of PPC case management seems unnecessary and availability between 8 am and 9 pm would be sufficient. However, parents may still feel greatly supported knowing that a team familiar with the situation of their child, is available during the evening and nights to guarantee continuous care. Future research should define the need and criteria for parents of availability of an adequate support system covering the needs between 9 pm and 8 am.

### Pro-active approach

About 25 % of the contacts are unplanned pro-actively initiated by the PPCT, and another 25 % of the contacts are initiated by parents (Fig. 1c). The majority of these unplanned contacts are with the specialized nurses (90 %). Less unplanned care is seen for the psychosocial disciplines, with 8 % pro-active contacts and 12 % initiated by parents, while 39 % of planned contacts are performed by these disciplines. This implies that the specialized nurses should be flexible and full-time available, to provide adequate response for the frequent unplanned contacts. The fact that parents initiated 25 % of all contacts, underlines the easy accessibility of the PPCT and indicates that parents feel confident and familiar enough with the PPCT to approach them.

### Topics discussed during contacts

Physical and psychosocial issues are discussed most frequently (23 and 38 % of all discussed topics respectively), with no difference between patients with MD and NMD.

Fifteen percent of the discussed topics covered socio-economic issues, which were significantly more often initiated by parents, and specifically, more by parents of children with NMD compared to MD (80/438 vs 20/234 p = 0.001). Why parents of NMD patients need more socio-economic information, could be explained by the more chronic course of disease, and the complex care they receive however there is no research supporting this assumption. More research on the differences in the needs and the appropriate palliative care for patients with MD and NMD is warranted. A more pro-active approach on socio-economic topics seems warranted in PPC management, which is in accordance with the information of Lindley et al. that the burden of costs of severely ill children has considerable impact on the family’s finances [25–28].

### Strengths and limitations

To our knowledge, this is the first description of the characteristics and content of case management in pediatric palliative care as provided by a multidisciplinary hospital-based PPCT. Focus was specifically on the discipline, the time-shift of contacts, initiation of contacts, and the topics discussed with parents. Since our PPCT supports children with any type of life shortening disease in care of our University Children’s hospital, insight is gained in differences in the needs for patients with MD and NMD. This knowledge provides opportunity to tune PPC with respect to the type of disease.

The completeness of response rate of questionnaires was checked via the routine administrative registration by the PPCT. From the 526 activities we received 455 completed questionnaires, indicating a response rate of at least 87 %. Since subsequent activities, such as telephone contact followed by e-mail contact, were registered as two contacts in the time registry, but were accompanied with one questionnaire, the actual response rate could in fact be even higher.

Due to an inadequate lay out of the form in 18 % of the questionnaires identification of the discipline was missing. However, 373 fully completed questionnaires describing 544 addressed topics were still available for analysis of the contacts per discipline.

In this study we did not address the appreciation of certain aspects of case management for patients, parents and the staff. This limits the conclusions we can draw from this study. For example, with our data, we might assume that integration of a chaplain may not be necessary since no consultation was performed. However, information on the contribution of the chaplain for staff support and education is needed before changes in the composition of the PPCT can be made. The many aspects of case management beyond individual care, such as the weekly multidisciplinary meetings, teaching and education, work meetings, coordination and team support were not the subject of this study [15]. It is estimated that around 60 % of the contracted time of case managers is spent on such activities and on time on duty, which content deserves further research in pediatric palliative care programs.

Also, for the suggestion that availability of the team from 8 am to 9 pm might be sufficient in the Dutch system with a strong primary health care, further research is needed to explore the value of 24-hour availability for parents.

In this study we did not look at patients’ individual support over the course of disease, but the full cohorts support during a set period. This may have biased the results, limiting the conclusions that can be drawn about the need of certain aspects of support. We do estimate that right after introduction of the PPCT and in the last days before a patient’s death, support may need to be more intensive, with the need of evening- and night- support, than in a stable phase of disease. Moreover, the kind of support needed, may be different during different phases of disease. Finally, the performance of care by our PPCT members might not be completely generalizable to other countries. Performance of PPC is highly dependent on the national and governmental organization of other components of care for children in a palliative setting, including aspects of home care, medical insurance coverage, geographical differences, and other factors such as religious beliefs, and family traditions. [10, 19, 29, 30].

## Conclusions

In conclusion this paper adds detailed insight in the content of pediatric palliative case management as provided by our hospital based multidisciplinary PPCT, which approaches parents pro-actively in 25 % of all contacts. We conclude that the multidisciplinary approach is especially needed for psychosocial, socio-economic and spare time issues. A more pro-active approach is still necessary for socio-economic topics. Since over half of the contacts are unplanned, our PPCT requires sufficient time for particularly the specialized nurses to work highly flexible to fulfill the needs of parents and patients. Easy accessibility and a flexible pro-active approach of the PPCT between 8 am and 9 pm seems to obviate the need for overnight availability of case management.

## Abbreviations

GP: General practitioner
IACP: Individual advance care plan
MD: Malignant disease
NMD: Non-malignant disease
PPC: Pediatric palliative care
PPCT: Pediatric palliative care team
